# Effectiveness of Chloral Hydrate on Brain MRI in Children with Developmental Delay/Intellectual Disability Comparing with Normal Intelligence: Single Tertiary Center Experience

**DOI:** 10.3390/children8121097

**Published:** 2021-11-29

**Authors:** Ja Un Moon, Ji Yoon Han

**Affiliations:** 1 Department of Pediatrics, College of Medicine, The Catholic University of Korea, Seoul 06591, Korea; gonicky@naver.com; 2 Department of Pediatrics, Seoul St. Mary’s Hospital, The Catholic University of Korea, Seoul 06591, Korea; 3 Department of Pediatrics, Daejeon St. Mary’s Hospital, The Catholic University of Korea, Daejeon 34943, Korea

**Keywords:** chloral hydrate, developmental delay, intellectual disability, sedative, children

## Abstract

Neurodiagnostic investigation requirements are expanding for diagnostic and therapeutic purposes in children, especially in those with developmental delay/intellectual disability (DD/ID). Thus, determination of optimal sedatives to achieve successful sedation and immobility without further neurological compromise is important in children with DD/ID. The purpose of this study is to assess the effectiveness and adverse reactions of chloral hydrate (CH) for brain magnetic resonance imaging (B-MRI) in children with DD/ID compared to those with normal intelligence (NI). We performed a retrospective chart review of children aged from 1 day to 12 years who required elective sedation using CH for B-MRI. About 730 cases (415 with DD/ID and 315 with NI) of CH sedation were conducted for B-MRI. Children with DD/ID showed a higher failure rate (22%) than did those with NI (6%); additional CH and prolonged sedation time were required. There was no difference in incidence of adverse reactions between DD/ID and NI groups (*p* = 0.338). Older or heavier children with DD/ID (*p* = 0.036 and *p* = 0.013, respectively), as well as those diagnosed with epilepsy or neuropsychiatric disorders showed higher risk of sedation failure (*p* < 0.001 for each). In conclusion, CH was a suboptimal sedative drug for children with DD/ID compared with those with NI. Other alternative or supplementary sedatives should be taken into consideration especially for those vulnerable groups.

## 1. Introduction

Developmental delay/intellectual disability (DD/ID) are common problems that affect between 1 and 3% of all pediatric populations [1]. Recently, neurodiagnostic investigations (e.g., brain magnetic resonance imaging (B-MRI), electroencephalography (EEG), and auditory brainstem response (ABR)) have played an important role in finding etiologic causes, predicting prognosis, and providing optimal treatment of many neurologic diseases. To obtain accurate data from neurodiagnostic procedures, it is important for the child to maintain absolute immobility during prolonged procedures. Pediatric patients, particularly those with DD/ID, frequently require sedatives to reduce motion artifacts, diminish anxiety, and successfully complete studies. Ideal sedative drugs to achieve adequate sedation effects should have minimal to no side effects, offer rapid onset times, have appropriate duration, and be available at low cost. However, sedation protocol based on children with DD/ID has not been well-established even though the incidence of side effects by sedation was found to be threefold higher than in children without DD/ID [2]. Chloral hydrate (CH), a non-opiate and non-benzodiazepine sedative hypnotic drug, has been used widely over the last two decades given its cost-effective profile and sedative-hypnotic potential [3]. CH is recommended for painless procedures in pediatric patients who have difficulty cooperating with neurodiagnostic investigations [3,4], but only a small number of studies has reported its use in pediatric patients with DD/ID. The purpose of this study was to evaluate the effectiveness and adverse effects of CH sedation for B-MRI in children with DD/ID to make rational, effective, and safe choices without causing further neurological exacerbation in those children.

## 2. Materials and Methods

We retrospectively reviewed the medical charts of pediatric patients who required sedation for B-MRI in the outpatient clinic. All pediatric patients aged from 1 day to 12 years who underwent elective sedation using CH at The Catholic University of Korea Seoul St. Mary’s Hospital from January 2010 to December 2020, were included. Patients who did not require sedation for imaging, who received other sedatives (e.g., midazolam, general anesthesia) rather than chloral hydrate, and who required sedation for MRI of body parts other than the brain were excluded. Data including demographics at the time of sedation (age, body weight, and gender), American Society of Anesthesiologists (ASA) score, B-MRI images, and diagnosis were collected. DD was defined as significant developmental delay (>2 standard deviations below age-matched peers) in two or more of the following areas: gross and fine motor skills, speech and language, cognition, personal and social skills, or activities of daily living in patients under the age of six years using Bayley Scales II or III edition. The deviation of a patient’s score from that of the normative mean is used to categorize developmental delay: normal, within 1 SD of the mean (≥85); mild, −1 SD to −2 SD (≥70 and <55). Intellectual disability is defined by an intelligence quotient (IQ) of 70 or below, with at least two behaviors related to adaptive functioning deficits manifesting before age 18 years. The severity of ID is classified into mild (IQ of 50–69), moderate (IQ of 35–49), severe (IQ of 20–34), and profound (IQ of <20) by the Wechsler intelligence test or Wechsler Preschool and Primary Scale of Intelligence.

All sedation processes followed the approved protocols of our institution. All children were monitored at the hospital from oral CH administration to complete recovery of consciousness. According to our protocols, the standard initial sedation consisted of oral chloral hydrate at a dose of 50 mg/kg (maximum dose of 1 g or 100 mg/kg). When satisfactory depth of sedation was not achieved after 30 min, a second dose of CH (dose: 25–50 mg/kg with a maximum dose of 1 g or 100 mg/kg) was given. Successful and effective sedation was considered to have been accomplished when clinically adequate B-MRI images were obtained after sedating the patients by the first or second administration of CH. To evaluate the safety of CH, we investigated the occurrence of adverse reactions. Once the child fell asleep, vital signs including heart rate, respiratory rate, and oxygen saturation were monitored every 15 min until the child fully awakened. Times from the first administration of CH to achieve adequate sedation (induction time, minutes), total sedation times for B-MRI scans (sedation duration, minutes), and times from arrival in the recovery room until fulfillment of the discharge criteria (recovery time, minutes) were investigated. All statistical analyses were conducted by SSPS software version 24.0 (IBM Corp., Armonk, NY, USA), and Chi-square or t-test were used for comparisons. We considered probability values less than 0.05 as statistically significant. This study was approved by the Institutional Review Board of The Catholic University of Korea (KC21RASI0764).

## 3. Results

### 3.1. Demographic Characteristics in Children with DD/ID or Normal Intelligence (NI)

In our study, a total of 1162 children were scheduled to undergo sedation for MRI during the study period. We excluded 469 patients based on the exclusion criteria detailed above, leaving 693 children were eligible for inclusion in the study and analysis. Of the 693 children, a total of 730 sedation procedures using CH (415 with DD/ID and 315 with NI) were performed for B-MRI. Among children with DD, speech and language delays were maximum (54%) followed by cognitive delay. Children delayed in more than one area were defined as global developmental delay (GDD), and those with GDD accounted for 21%. Table 1 presents the comparison of baseline characteristics, sedation data, and outcomes between children with DD/ID and NI. The mean age at sedation in children with DD/ID was significantly higher (39 months, range 2 days to 12 years) than in children with normal intelligence (31 months, range 7 days to 11 years) (*p* = 0.011), and the mean body weight was significantly heavier in children with DD/ID than in NI (17 kg and 12 kg, respectively, *p* = 0.035). Children with DD/ID tended to have higher ASA scores than did those with NI, but no significant difference was found between the two groups (*p* = 0.322). Although B-MRI analyses showed abnormalities were more common in DD/ID patients (*n* = 122, 29%) compared to patients with NI (*n* = 40, 13%), there was no statistical significance. In children with DD/ID, structural abnormalities (e.g., lissencephaly, schizencephaly) accounted for a large proportion in abnormal results of B-MRI while most children with NI who had abnormal B-MRI results showed incidental findings (e.g., arachnoid cyst, mega cisterna magna). The mean initial dose (51.7 mg/kg and 51.2 mg/kg, respectively, *p* = 0.563) and the mean additional dose (36.2 mg/kg and 34.8 mg/kg, respectively, *p* = 0.373) of chloral hydrate did not differ between the two groups. More children (21%) with DD/ID failed to experience adequate sedation, requiring additional doses of CH, compared to those of NI (*p* < 0.001). In both groups, there was a trend of increasing need for additional dose of CH with an increase in age (*p* = 0.009 in children with DD/ID and *p* = 0.023 in NI).

**Figure 1:**
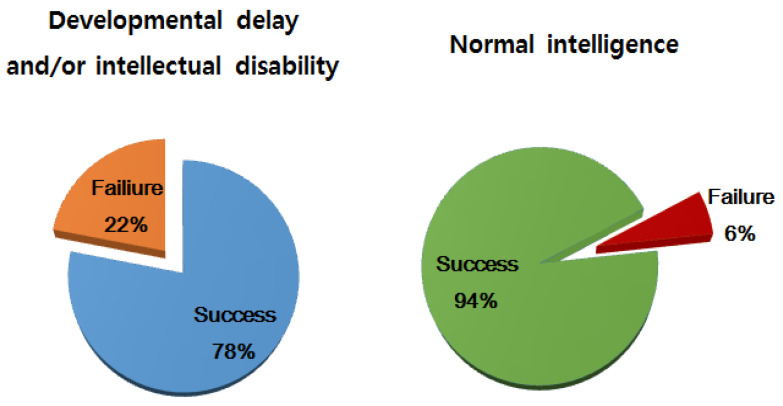
Effectiveness of chloral hydrate in the two groups (*p* = 0.047).

Successful sedation rate of CH for B-MRI was 94% in developmentally normal children but was significantly lower in children with DD/ID (Figure 1). The sedation time for B-MRI was significantly longer in children with DD/ID (53 min) than in children with NI (29 min, *p* < 0.001), while no differences in induction and recovery time were found. Among the total 730 sedations, 49 (7%) showed adverse reactions. No life-threatening or serious adverse reactions (e.g., requiring ventilation or hospitalization) were reported in the two groups during the period of sedation. The most frequently observed adverse reaction in both groups was nausea/vomiting (*n* = 23, 3%), followed by respiratory oxygen desaturation (*n* = 18, 2.5%) that resolved completely with or without an additional administration of 100% oxygen, irritability/hyperactivity (*n* = 5, 0.7%), and bradycardia that resolved spontaneously (*n* = 3, 0.4%). Children with or without DD/ID who required additional CH showed greater respiratory oxygen desaturation demanding supplemental oxygen (*p* = 0.031 and *p* = 0.047, respectively). However, there was no statistically significant difference in frequency of adverse reactions between the two groups (*p* = 0.338).

### 3.2. Demographics of Sedation by Chloral Hydrate in Success and Failure Groups with Developmental Delay/Intellectual Disability

We also compared demographics between the successful sedation group (*n* = 323, 78%) and the failure group (*n* = 92, 22%) in children with DD/ID (Table 2). The success or failure to sedate did not correlate with gender (*p* = 0.544), grade of impairment (*p* = 0.450), ASA score (*p* = 0.322), or mean initial or additional dose of CH (*p* = 0.214 and *p* = 0.117, respectively). However, children aged more than 2 years and greater than 20 kg in weight were significantly more frequent in the failure group of DD/ID (*p* = 0.036 and *p* = 0.013, respectively). Abnormal results of B-MRI presented at a higher rate in the failure group (48%), whereas 24% presented in the successful group (*p* = 0.029). Children with DD/ID who were diagnosed with epilepsy and neuropsychiatric disorders were significantly more frequent in the failure group (*p* < 0.001 for each), whereas children who were diagnosed with hearing or vision impairments were more frequent in the success group (*p* = 0.004). Behavioral disorders accounted for most portions of children with neuropsychiatric disorders in our study including autism spectrum disorder and attention deficit hyperactivity disorder. There were no significant differences in other diagnoses between the two groups.

## 4. Discussion

The purpose of sedation is to reduce physical and mental discomfort, including anxiety. Sedation in children is different from that in adults in that it modifies behavior (immobility) while also relieving discomfort and anxiety. Thus, inadequate sedation can lead to an unsuccessful procedure, as well as disheartening effects on family and caregivers. American Society of Anesthesiologists has defined a four-level scale of sedation depth in children: (1) minimal sedation (anxiolysis), (2) moderate sedation (conscious sedation), (3) deep sedation, and (4) general anesthesia [5]. Pediatric sedation protocols have been modified according to length of sedation time, age of the patients, type of procedures, and institutional preferences. B-MRI is a useful, noninvasive, and radiation-free diagnostic procedure especially used in patients with neurologic disease. Nevertheless, it is always an obstacle for the patient to remain motionless for optimal image quality when the patient is younger than 6 years old or has DD/ID, in which case deep sedation is often required.

General anesthesia provides the best motionless state during a procedure, but it is invasive and expensive compared to CH sedation. For non-painful procedures without intravenous access, oral routes are more comfortable and less invasive. CH is a generally well tolerated, easy to administer, and inexpensive sedative/hypnotic agent without analgesic characteristics and has been used as a light sedative for children in various procedures outside the operating room for two decades [6]. Its mechanism of action is mediated by agonistic effects of gamma-aminobutyric acid (GABA) type A receptors in the central nervous system [7]. It is highly lipid-soluble, which facilitates entry into the brain and rapid onset of sedative effect [8]. The onset of action occurs within 10–15 min, with peak concentration within 30 min and the duration of action varying from 60 to 120 min [9,10]. The usual dose of CH is about 50–100 mg/kg with a maximum dose of 2 g [11,12]. Successful CH sedation rate of 88–99% was achieved with an initial dose of 60–100 mg/kg and 94.2% success rate was achieved with a single mean dose of 77.5 mg/kg in previous reports [13,14]. Even at a relatively low dose of CH, the sedation rate reached 90%; after administration of an additional dose of CH, the sedation rate reached 99% [15]. In this study, although the mean initial and additional CH doses were similar in each group, the failure rate was significantly higher in children with DD/ID (78%) compared with NI (94%).

Since children have different physiological characteristics depending on age and body weight, their responses to sedative agents can vary. Many studies have reported the response of CH sedation with or without other sedatives, and their success rates varied considerably by age, weight, and underlying disease (Table 3). 

Our successful sedation rate in children with NI was 94%, which is similar to the results given in previous reports, which ranged from 86% to 97.7% [11,12,16,17]. Mataftsi et al. had demonstrated the efficacy of CH by achieving successful sedation in from 88% to 99% of pediatric ophthalmology patients [18]. Even though CH is an effective drug especially in children under 48 months undergoing painless procedures [10], children with neurodevelopmental disorders showed decreased efficacy and increased incidence of adverse reactions compared with developmentally normal children [12]. Children with DD/ID have difficulty in sedation due to limited attention, baseline hyperactivity, poor communication, or exaggerated reactions to environmental changes [19]. Nonetheless, only a few studies associated with sedation have enrolled patients with DD/ID [2,20,21]. In our study, children with DD/ID accounted for more than 50% of the patient population, and their successful sedation rate was 78%. Cortellazzi et al. reported about 2.3% failure rate with or without other sedatives in combination with CH in children with neurological disorders who underwent B-MRI [12]. Another study showed only 0.4% failure rate using CH with pentobarbital and fentanyl for B-MRI [20]. Our higher failure rate than those previous studies might result from (1) use of CH as the sole sedative (initial and additional) in this study compared to others, (2) initial and additional doses of CH were lower than in other studies because the protocol in our institution includes an initial CH dose of 50 mg/kg followed by an additional dose of 25–50 mg/kg if the child fails to sleep, (3) a higher treatment failure rate in older children [11], and a large number of older children with DD/ID might contribute to increased failure rate. Sedation duration in children with DD/ID was longer than in normal children, whereas no difference was shown in induction or recovery time. We hypothesized that, since children with DD/ID required additional CH, the sedation duration was extended accordingly. Some studies revealed risk factors for sedation failure as age, weight, ASA physical status, cognitive impairment, and other neurological disorders [12,17,22,23]. We observed that older age and heavier body weight, abnormalities in B-MRI, and diagnosis of epilepsy or neuropsychiatric disorders played a role in sedation failure in children with DD/ID. One report also suggests that children’s weight was a risk factor for CH sedation failure, but that the type of neurological disease was not [12]. Similar to previous report, our study suggested that older or heavier children are prone to CH sedation failure [17]. In children older than 2 years or with body weight in excess of 20 kg, sedation requires alternatives to CH regardless of DD/ID or NI.

Safe sedation for diagnostic and therapeutic procedures in children requires a systemic approach including evaluation of underlying medical conditions, appropriate fasting, airway examination, and adequate monitoring during and after procedures [24]. Infants who require deep sedation to maintain a motionless state during the B-MRI procedure have a higher incidence of cardiopulmonary adverse effects that can lead to death [25], but CH appears to have relatively little effect on cardiopulmonary systems [26,27]. CH has disadvantages such as narrow therapeutic index, inter-individual variability, prolonged sedation and recovery times, paradoxical reaction, and inconsistent sedative effects [4,22]. Adverse reactions from CH were rash, gastric irritation, diarrhea, nausea/vomiting, decreased oxygen saturation, prolonged sedation, hypotension, and hyperactivity/irritability/anxiety [15,28]. Despite few fatal adverse effects on the cardiopulmonary system, irritation to the gastric mucosa can lead to aspiration of stomach materials, which can be fatal in sedated children. In contrast to a previous study, the incidence rate of adverse reactions was not significantly increased in children with DD/ID in this study, although the use of additional CH was more frequent in those with DD/ID [20]. Common adverse reactions associated with CH were nausea and vomiting, followed by agitation and oxygen desaturation, as in other reports [11,28]. Although a hypoxic adverse event related to CH was more likely to develop in children with DD, those with congenital heart disease or respiratory disease as an underlying condition were more prone to respiratory oxygen desaturation regardless of developmental state [20]. Even though the incidence of adverse reactions in children with DD was slightly lower than that reported by other authors using CH alone or in combination with other sedatives, it cannot be concluded to be safe given that it is 6–7% as a single sedative with no existing antidote [12,20]. As described above, CH has shown favorable outcomes and some side effects in children. The Cochrane Review suggests that CH is an effective sedative agent with similar sedation failure rate compared with those of oral dexmedetomidine, hydroxyzine, oral midazolam, and clonidine and a more effective sedative agent with a lower sedation failure rate compared with promethazine [3]. While most of the included studies showed that CH was safe with no increase in adverse reactions compared to other sedative agents, the Food and Drug Administration and the European Medicines Agency have partially withdrawn their approval for CH due to potential carcinogenic and genotoxic risks [29]. However, CH is used in many countries and has been for more than 100 years for dental, ophthalmic, or otolaryngological tests, and there are no definitely safer alternatives.

We showed epilepsy and neuropsychiatric disorders such as autism spectrum disorder or attention deficit hyperactivity disorder to be risk factors for CH sedation failure in children with DD/ID. These results are similar to other reports suggesting that children with neuropsychiatric disorders are difficult to sedate with CH and have increased incidence of adverse reactions [19,30], suggesting that underlying disease and comorbidity should be considered when deciding whether to use CH as a sedative for B-MRI. 

Although CH seemed to be an adequate sedative agent for developmentally normal children and especially those younger and lighter in weight due to its adequate efficacy with relatively low adverse reaction rates, our study could not prove CH to be safe and effective in children with DD/ID. Currently, dexmedetomidine (intranasal, intramuscular, or intravenous) is preferred for uncooperative children or those with cognitive impairments, especially for noninvasive procedural sedation providing fewer cardiorespiratory adverse reactions with high effectiveness via various routes of administration [31,32,33,34]. Other sedatives including hydroxyzine and melatonin are used as common supplements for sedation in combination with CH and show favorable outcomes compared to CH alone [3,35,36,37]. For these reasons, the use of supplements such as hydroxyzine, melatonin, or alternative sedatives in conjunction with dexmedetomidine should be considered especially in children with epilepsy or neuropsychiatric problems with DD/ID for B-MRI sedation. Given that abnormalities in B-MRI correlated with high failure rate, for those with DD/ID and suspected to have abnormalities or who were confirmed to have abnormalities on B-MRI, addition of supplements initially or use of alternative sedatives should be considered due to the lower effectiveness of CH alone. Since it is difficult to maintain an intravenous line in children with DD/ID, more effective and safe oral sedative drugs are needed for this vulnerable group.

The limitations of this study were its small sample size and the retrospective nature of chart review. Adverse reaction rate might have been underestimated because they were noted by different nurses and physicians. Minor reactions (nausea, transient oxygen desaturation) might have been overlooked in both groups. Therefore, large prospective studies are needed to identify the safety and effectiveness of CH in pediatric patients with DD/ID and to suggest alternative sedative agents.

## 5. Conclusions

In summary, CH as the sole drug seems to be a suitable sedative with a higher success rate for developmentally normal children undergoing B-MRI sedation. However, children with DD/ID showed difficult and suboptimal sedation, required more frequent additional CH, and had prolonged sedation time. Since children with DD, and especially those diagnosed with epilepsy or neuropsychiatric disorders, have higher failure rates for CH sedation, we recommend using other alternatives or additional drugs such as melatonin or hydroxyzine. Further studies are needed to determine the optimal sedation protocol that will achieve favorable outcomes and minimize adverse reactions in the DD/ID group.

## Figures and Tables

**Table 1:** Demographics of sedation in children with developmental delay/intellectual disability and normal intelligence.

Variables	DD/ID (*n* = 415)	NI (*n* = 315)	*p* Value
Gender, n (%)			0.335
Male	249 (60%)	173 (55%)	
Female	166 (40%)	142 (45%)	
Mean age, month (±SD)	39 (±30.5)	31 (±28.4)	0.011
Under 2 years, n (%)	200 (48%)	205 (65%)	
Over 2 years, n (%)	216 (52%)	110 (35%)	
Mean weight, kg	17 (±5.5)	12 (±4.1)	0.035
Under 20 kg, n (%)	336 (81%)	277 (88%)	
Over 20 kg, n (%)	79 (19%)	38 (12%)	
ASA score			0.273
I	292 (70%)	268 (85%)	
II	123 (30%)	47 (15%)	
III	0 (0%)	0 (0%)	
Brain MRI			0.087
Normal	288 (70%)	271 (86%)	
Abnormal	122 (29%)	40 (13%)	
Missing data	5 (1%)	4 (1%)	
Mean initial dose of chloral hydrate,	52 (±6.88)	51 (±5.9)	0.563
mg/kg (±SD)			
Use of additional chloral hydrate, n	87 (21%)	32 (10%)	<0.001
Mean additional dose of chloral hydrate,	36 (±11.5)	35 (±11.3)	0.373
mg/kg (±SD)			
Outcome			0.047
Fail	92 (22%)	19 (6%)	
Success	323 (78%)	296 (94%)	
Induction time, minutes (±SD)	34 (±13)	33 (±17)	0.445
Sedation duration, minutes (±SD)	53 (±21)	29 (±9)	<0.001
Recovery time, minutes (±SD)	27.7 (±29.0)	28.1 (±18.5)	0.188
Adverse reactions, n (%)	30 (7%)	19 (6%)	0.338

DD, developmental delay; ID, intellectual disability; NI, normal intelligence; SD, standard deviation; ASA, American society of anesthesiologists; MRI, magnetic resonance imaging.

**Table 2:** Demographics of sedation in the successful and failure groups with developmental delay/intellectual disability.

Variables	Success	Failure	*p* Value
	*n* = 323	*n* = 92	
Gender, n (%)			0.544
Male	192 (59%)	57 (62%)	
Female	131 (41%)	35 (38%)	
Mean age, month (±SD)	38 (±28.9)	65 (±31.4)	0.036
Under 2 years, n (%)	157 (53%)	16 (19%)	
Over 2 years, n (%)	142 (47%)	68 (81%)	
Mean weight, kg	13.7	21.4	0.013
Under 20 kg, n (%)	284 (88%)	50 (54%)	
Over 20 kg, n (%)	39 (12%)	42 (46%)	
Grade of impairments			
Mild	155 (48%)	40 (44%)	0.45
Moderate	87 (27%)	29 (31%)	
Severe	81 (25%)	23 (25%)	
ASA score			
I	229 (71%)	63 (69%)	0.322
II	94 (29%)	29 (31%)	
III	0 (0%)	0 (0%)	
Mean initial dose of chloral hydrate,	51 (±6.9)	49 (±10.3)	0.215
mg/kg (±SD)			
Mean additional dose of chloral hydrate,	35.4 (±10.8)	36.1 (±11.0)	0.117
mg/kg (±SD)			
Brain MRI			0.029
Normal	242 (75%)	46 (50%)	
Abnormal	78 (24%)	44 (48%)	
Missing data	3 (1%)	2 (2%)	
Cause of DD/ID or combined disorders			
Epilepsy	55 (17%)	38(41%)	<0.001
Hearing or vision impairments	35 (11%)	2 (2%)	0.004
Neuropsychiatric disorders	26 (8%)	32 (35%)	<0.001
Neuromuscular disease	6 (2%)	4 (4%)	0.407
Cerebral palsy	29 (9%)	11 (12%)	0.377
Cerebrovascular disease	32 (10%)	7 (8%)	0.204
Genetic disorders	39 (12%)	10 (10%)	0.551
Adverse reactions	21 (9%)	9 (9%)	0.442

DD, developmental delay; ID, intellectual disability; NI, normal intelligence; SD, standard deviation; ASA, American society of anesthesiologists; MRI, magnetic resonance imaging.

**Table 3:** Efficacy and adverse reactions of chloral hydrate in pediatric patients during recent decades.

Journal (Year)	Procedure	Number of Cases	Ages	Body Weight (Kg)	Dose (mg/kg)	Efficacy (%)	Adverse Reactions (%)	Remarks
Necula et al. (2019)	ABR	323	MA: 28.18 ± 18.10 (months)	NA	MD: 0.75 mL/kg	94.1	20.5	
Valenzuela et al. (2016)	ABR	725	NA	NA	MD: 52	95.9	19.2	
Reynolds et al. (2016)	ABR	41	Median: 25.6 (months)	Median: 12.8	NA	66	0	Comparing with intranasal dexmedetomidine
Stephan et al. (2015)	ABR	41	MA: 2.78 ± 1.33 (years)	MW: 12.41 ± 3.61	NA	95	NA	Comparing with intranasal midazolam
Finnemore et al. (2013)	MRI	411	Median: 37 (weeks)	Median: 3.6	Median: 50	NA	5.1	Neonates
Lee et al. (2012)	MRI	399	MA: 24.9 ± 19.0 (months)	MW: 11.7 ± 4.5	MD: 61.5 ± 22.1	91.5	12.5	
Delgado et al. (2015)	MRI	7103	Median: 2.5 (years)	NA	MD: 57.14	95.2	1.8	
Cortellazzi et al. (2007)	MRI	1104	MA: 30.0 ± 18.8 (months)	MW: 12.6 ± 4.9	MD: 86.3 ± 9.9	97.7	7.6	Neurologically impaired children
Zhang et al. (2016)	MRI	40	MA: 3.8 ± 1.5 (months)	MW: 6.1 ± 1.6	NA	80	NA	Rescue sedation by additional chloral hydrate vs. intranasal dexmedetomidine
Malviya et al. (2004)	MRI	35	MA: 4.2 ± 1.7 (years)	NA	MD: 71.9 ± 11.1	97	22	Comparing with pentobarbital
Bracken et al. (2012)	MRI	653	MA: 15 (months)	MW: 10.1	MD: 46.2 (infant), 66.2 (>1 year)	96.7	0.3	Including ultrasonogram, computed tompgraphy, or nuclear medicine tests
West et al. (2013)	OPT tests	1509	NA	NA	MD: 945 mg	96.69	7.89	
Chan et al. (2017)	OPT procedure	153	NA	NA	NA	94.1	NA	
Wilson et al. (2014)	OPT procedure	380	NA	MW: 10.6 ± 3.4	MD: 77.5	97.9	0.26	
Wandalsen et al. (2016)	Pulmonary function test	277	NA	NA	Median: 70	93.5	6.5	Infants
Sezer et al. (2013)	Sleep EEG	141	MA: 6.4 ± 1.3 (years)	NA	NA	98	5	Comparing with hydroxyzine

ABR, auditory brainstem response; MRI, magnetic resonance imaging; EEG, electroencephalogram; OPT, ophthalmologic; MA, mean age; MD, mean dose; MW, mean weight, NA: not available.

## Data Availability

The data presented in this study are available upon request from the corresponding author.
